# *KRAS* Sequence Variation as Prognostic Marker in Patients With Young- vs Late-Onset Colorectal Cancer

**DOI:** 10.1001/jamanetworkopen.2023.45801

**Published:** 2023-11-30

**Authors:** Mayada A. Aljehani, Jeffrey Bien, Jerry S. H. Lee, George A. Fisher, Albert Y. Lin

**Affiliations:** 1 Ellison Institute of Technology, Los Angeles, California; 2 Stanford University School of Medicine, Stanford, California; 3 Department of Medicine, Keck School of Medicine, University of Southern California, Los Angeles; 4 Department of Chemical Engineering and Material Sciences, Viterbi School of Engineering, University of Southern California, Los Angeles; 5 Department of Quantitative and Computational Biology, Dornsife College of Letters, Arts and Sciences, University of Southern California, Los Angeles; 6 Division of Oncology, Department of Medicine, VA Palo Alto Medical Center, Palo Alto, California

## Abstract

**Importance:**

The understanding of the association between *KRAS* sequence variation status and clinical outcomes in colorectal cancer (CRC) has evolved over time.

**Objective:**

To characterize the association of age at onset, tumor sidedness, and *KRAS* sequence variation with survival among patients diagnosed with CRC.

**Design, Setting, and Participants:**

This cross-sectional study used data extracted from the Surveillance, Epidemiology, and End Results database. Patients diagnosed with adenocarcinoma of the colon or rectum from 2010 through 2015 were included and were classified as having young-onset (YO) cancer if diagnosed between ages 20 to 49 years and late-onset (LO) cancer if diagnosed at age 50 years or older. Data were analyzed from April 2021 through August 2023.

**Main Outcomes and Measures:**

CRC cause-specific survival (CSS) was summarized using Fine and Gray cumulative incidence and Kaplan-Meier curves. Estimation of subdistribution hazard ratios (sHRs) for the association of *KRAS* status, age at onset, and tumor location with CRC CSS was conducted using the Fine and Gray competing risk model. Cox proportional hazards regression was used to estimate and compare HRs.

**Results:**

Among 21 661 patients with *KRAS* sequence variation status (mean [SD] age at diagnosis, 62.50 [13.78] years; 9784 females [45.2%]), 3842 patients had YO CRC, including 1546 patients with *KRAS* variants, and 17 819 patients had LO CRC, including 7311 patients with *KRAS* variants. There was a significant difference in median CSS time between patients with variant vs wild-type *KRAS* (YO: 3.0 years [95% CI, 2.8-3.3 years] vs 3.5 years [95% CI, 3.3-3.9 years]; *P* = .02; LO: 2.5 years [95% CI, 2.4-2.7 years] vs 3.4 years [95% CI, 3.3-3.6 years]; *P* < .001). Tumors with variant compared with wild-type *KRAS* were associated with higher risk of CRC-related death (YO: sHR, 1.09 [95% CI, 1.01-1.18]; *P* = .03; LO: sHR, 1.06 [95% CI, 1.02-1.09]; *P* = .002). Among patients with YO cancer, mortality hazards increased by location, from right (sHR, 1.02 [95% CI, 0.88-1.17) to left (sHR, 1.15 [95% CI, 1.02-1.29) and rectum (sHR, 1.16 [95% CI, 0.99-1.36), but no trend by tumor location was seen for LO cancer.

**Conclusions and Relevance:**

In this study of patients diagnosed with CRC, *KRAS* sequence variation was associated with increased mortality among patients with YO and LO tumors. In YO cancer, variant *KRAS–*associated mortality risk was higher in distal tumors than proximal tumors.

## Key Points

**Question:**

How does the prognostic profile of *KRAS* sequence variation compare between patients with young-onset and late-onset colorectal cancer?

**Findings:**

In this cross-sectional study of 21 661 patients with colorectal cancer, *KRAS* sequence variation was associated with worse survival compared with *KRAS* wild type in patients with young- and late-onset cancer. The median cause-specific survival for *KRAS* variant vs *KRAS* wild type was 3.0 and 3.5 years in young-onset and 2.5 vs 3.4 years in late-onset cancer.

**Meaning:**

These findings may provide additional clarity into the association between *KRAS* sequence variants and clinical outcomes and between *KRAS* status and age of colorectal cancer onset.

## Teaser

This cross-sectional study investigates the association of *KRAS* sequence variation with survival among patients with young- vs late-onset colorectal cancer.

## Introduction

Colorectal cancer (CRC) remains a frequently occurring and deadly disease in the US, with new presentation in approximately 153 000 people annually and more than 52 000 expected annual deaths in 2023.^1^ It is the third most common cancer in the US and the second leading cause of cancer-related death behind lung cancer. Nevertheless, the overall mortality has declined annually since 1990, attributable in part to the advent of screening modalities and improvement in treatments.

In contrast to this overall decline in mortality, there was a recent increase in CRC incidence and deaths among adults diagnosed at younger than age 50 years (young-onset [YO] CRC).^2,3^ Moreover, some unique features among patients with YO CRC garnered attention, emphasizing distinct demographic, clinical, histologic, and molecular profiles compared with patients with CRC onset at age 50 years or older (late-onset [LO] CRC) in several notable ways.^4^ Clinically and histologically, patients with YO CRC had a higher risk of poorly differentiated tumors and anaplastic tumors and displayed mucinous and signet ring cell histology.^2^ These tumors were also more likely to present more distally and at advanced stages. As a consequence of these features, in 2020 the American Cancer Society lowered the recommended age to start screening from 50 to 45 years for individuals at the mean level of risk.^5^ Despite these clinical, histopathologic, and molecular associations, the underlying cause of the increasing incidence remains unknown.^6^

In a 2019 study wherein 18 218 tumor samples underwent next-generation sequencing, Lieu et al^7^ found that among patients with tumors exhibiting microsatellite stability, those with YO CRC (uniquely defined as cancer diagnosed at age <40 years in this study) differed from those with LO CRC, with a lower frequency of *APC*, *KRAS*, *BRAF*, and *FAM123B* sequence variants and a higher frequency of *TP53* and *CTNNB1* variants. Among patients with microsatellite-stable cancer, this analysis found a statistically significant difference in incidence of *KRAS* variants, with 52.4% among LO and 45.6% among YO tumors.^7^ In 2019, Willauer et al^4^ characterized YO cancers into unique molecular subtypes by analyzing 36 000 patient samples from 4 combined cohorts. Their analysis also demonstrated a numerically lower but statistically nonsignificant incidence of *KRAS* variants among patients with YO cancer.^4^ In contrast, Watson et al^8^ in 2016 reported a higher incidence of *KRAS* variants in YO cancers (also defined as diagnosis at age <40 years), although this finding was compromised by a smaller sample size.

Of common genetic alterations seen in CRC, *KRAS* variants were of particular interest because of their frequency (approximately 50% incidence in metastatic CRC) and because they carry treatment implications; their presence was associated with resistance to treatment with anti–epidermal growth factor receptor medications, such as panitumumab and cetuximab.^9,10^ Whether a *KRAS* variation was independently associated with prognostic mortality profiles had been a complex and controversial topic that evolved over decades with increased testing availability. Specifically, the presence of *KRAS* variants in CRC had been theorized to independently associate with adverse outcomes. However, we hypothesize that this association may depend on other variables, such as the specific sequence variation, tumor location, and possibly age of onset.

There were 2 early large clinical trials, the 1998 Kirsten Ras Mutations in Patients With Colorectal Cancer (RASCAL) study^11^ and 2001 RASCAL II study,^12^ that were among the first to demonstrate the prognostic role of *KRAS* sequence variation in CRC. Whereas the RASCAL study found an association between *KRAS* variants and adverse prognosis, the results of the RASCAL II study suggested that this adverse prognosis was limited to a specific glycine-to-valine substitution in *KRAS* codon 12. There were 2 later studies, the 2009 Cancer and Leukemia Group B (CALGB) 89803 trial^13^ and 2010 Pan-European Trial in Adjuvant Colon Cancer (PETACC)-3,^14^ that were then unable to replicate a link between *KRAS* variant status and clinical outcomes. Recently, multiple prospective studies in CRC had again demonstrated varying degrees of prognostic significance of a *KRAS* variant. Imamura et al in 2012^15^ found that sequence variations in *KRAS* codon 12 but not codon 13 were associated with inferior survival. The PETACC-8 trial in 2014^15^ found that *KRAS* sequence variations were associated with shorter time to relapse, and a subset analysis^16^ found that the linkage persisted only for sequence variations of codon 12 and (similar to the results of Imamura et al^15^) not codon 13; this trial further specified that survival implications of the variation were sustained only in distal, stage III tumors. However, Yoon et al in 2014^17^ linked shorter disease-free survival after resected stage III colorectal tumors to sequence variations in *KRAS* codon 12 or 13, as did Modest et al in 2016^18^ in the metastatic setting. Finally, Taieb et al in 2017^19^ demonstrated that any *KRAS* variant among patients with stage III, microsatellite-stable disease connoted worse clinical outcomes.

In addition to the specific genomic sequence variation, tumor site was explored as a potential linkage with *KRAS* status. For instance, the North Central Cancer Treatment Group (NCCTG)/Alliance N0147 trial in 2014^20^ demonstrated that *KRAS* sequence variation was more likely to occur proximally, a finding strengthened in a 2015 analysis of the same trial demonstrating that *KRAS* variants in tumors occurring distally were independently associated with mortality. This unique association of tumor location, *KRAS* status, and death was in turn confirmed in a population-based study by Charlton et al in 2020.^21^

As highlighted by Lieu et al^7^ previously, the prevalence of these variations can vary by age of onset. Therefore, distinctions in *KRAS* sequence variation rates between YO and LO cancers may carry complex implications for prognosis, treatment strategies, and perhaps even screening recommendations.^7^

In this study, our objectives were to assess the interplay among age at CRC onset, tumor location, and *KRAS* variant status and their collective association with CRC survival time and mortality using a population-based data set. While previous studies have explored the prevalence of *KRAS* variants in CRC, to our knowledge, our research was the first to delve into the intersection of age of onset, tumor location, and *KRAS* sequence variation status.

## Methods

The Veterans Administration institutional review board granted this cross-sectional study review exemption status because it was not human participant research and waived patient consent because the data were retrieved from a public database (Surveillance, Epidemiology, and End Results [SEER] Research Data). Reporting of study results followed the Strengthening the Reporting of Observational Studies in Epidemiology (STROBE) reporting guideline for cross-sectional studies.

### Data Source and Study Population

Data were derived from the SEER database of the National Cancer Institute, a population-based cancer database covering more than 25% of the US population. Using the case listing session of the SEER*Stat statistical software version 8.3.4, we queried the SEER 18 registries 2010 to 2015 data set^22^ and obtained individual patient demographic, tumor characteristic, site-specific factor (SSF)-9 (*KRAS* status, obtained via special request), and survival data. YO was defined as age at diagnosis 20 years or older and younger than 50 years. LO was defined as age at diagnosis 50 years or older. Patients were included in the analysis if they had pathologically confirmed diagnosis of CRC. We identified CRC diagnoses using *International Classification of Diseases for Oncology* (*ICD-O*). Colon cancer diagnoses were retrieved using topographic codes C18.0 to C18.9, while C20.9 was used for rectal cancer. Histology codes 8000/3, 8010/3, 8020/3, 8140/3, 8143/3, 8144/3, 8145/3, 8210/3, 8211/3, 8220/3, 8221/3, 8255/3, 8260/3, 8261/3, 8262/3, and 8263/3 were classified as adenocarcinoma, while 8213/3, 8480/3, 8481/3, 8490/3 were classified as other.^23^ Patients with age less than 20 years (64 patients), benign tumors (7376 patients), histologic type other than standard adenocarcinoma (2480 patients), incomplete cause of death or follow-up information (2810 patients), or diagnosis confirmed by autopsy or death certificate only (1560 patients) were excluded from this study. Access to CRC SSF-9 was approved and provided by SEER.

### Study Outcomes

Cause-specific survival (CSS) for CRC death in the presence of competing risk of non-CRC death was the primary study end point. It was calculated as time from diagnosis to CRC-related death or study end, December 31, 2015, whichever came first, with non-CRC death as a competing risk. Additionally, CSS served as a secondary end point with no consideration for competing event and was calculated as time from diagnoses to CRC-related death or study end, December 31, 2015, whichever came first. Vital status and SEER cause of death classification variables were used to identify CSS. The maximum follow-up time for this study was 71 months.

### Study Covariates

Patient demographic and clinical variables included age at diagnosis, race and ethnicity, year at diagnosis, sex, and insurance status. Race and ethnic classification were based on patient information extracted from medical records. Categories according to SEER classification were Hispanic, non-Hispanic American Indian or Alaska Native, non-Hispanic Asian or Pacific Islander, non-Hispanic Black, and non-Hispanic White. In this study, the American Indian and Alaskan Native category was combined with unknown race and ethnicity due to small numbers. When the information was missing, SEER used algorithmic imputation.^24^ Tumor characteristics included sidedness, TNM stage according to the 7th edition of the American Joint Committee on Cancer staging system,^25^ histology, histopathologic grade, and *KRAS* status. Tumors with *ICD-O-3* topography codes C18.0 (cecum), C18.2 (ascending colon), C18.3 (hepatic flexure of colon), and C18.4 (transverse colon) were classified as right sided, and those with codes C18.5 (splenic flexure of colon), C18.6 (descending colon), C18.7 (sigmoid colon), and C19.9 (rectosigmoid) were classified as left sided. Additionally, rectal cancer tumors were identified using topographic code C20.9. All covariates were selected a priori based on biological or sociodemographic significance and were included in each analysis as appropriate.

### Statistical Analysis

Differences in demographic, clinical, and pathologic characteristics between YO vs LO cancer or wild-type vs variant *KRAS* were assessed using the *t* test for continuous variables and χ^2^ tests for categorical variables. In the presence of non-CRC competing death, the Fine and Gray^26^ model was used to assess *KRAS* variation, age, and tumor location as prognostic factors associated with CSS. In addition, Fine and Gray and Gray tests were used to model and compare cumulative incidence of CRC death. We regressed the subdistribution hazard ratio (sHR) of CRC death on covariates described previously. Similar analyses for cumulative incidence of non-CRC death were also conducted. Separate comparisons were conducted for the median CSS between variant vs wild-type *KRAS* in LO and YO groups using Kaplan-Meier curves and the log-rank test. Additionally, Cox proportional hazard regression models were used to estimate mortality HRs with 95% CIs. To minimize differences due to age and location, subgroup analyses were conducted within LO and YO groups and each tumor location group. In addition, to assess the robustness of our results, stratified analyses by stage were conducted. All tests of statistical significance were 2-sided and conducted at a significance level of α = .05, using R statistical software version 4.3.0 (R Project for Statistical Computing). Data were analyzed from April 2021 through August 2023.

## Results

### Demographic Characteristics

Among 202 237 total patients with CRC in the database, there were 21 661 patients for whom *KRAS* testing results were available and who were included in the analysis (mean [SD] age at diagnosis, 62.50 [13.78] years; 9784 females [45.2%]; 2471 Hispanic [11.4%], 252 non-Hispanic American Indian, Alaskan Native, or unknown [1.2%], 1683 non-Hispanic Asian or Pacific Islander [7.8%], 2767 non-Hispanic Black [12.8%], and 14488 non-Hispanic White [66.9%]). The Table shows the distribution of demographics, clinical characteristics, and outcomes by YO vs LO group in the overall population. There were 3842 patients with YO CRC (mean [SD] age, 41.97 [6.25] years), including 1546 patients with *KRAS* variants, and 17 819 patients with LO CRC (mean [SD] age, 66.93 [10.57] years), including 7311 patients with *KRAS* variants. In the YO group, variant *KRAS* was more prevalent in females (807 females [52.2%] vs 739 males [47.8%]; *P* < .001), while in the LO group, a lower proportion of females (5824 females [55.4%] vs 3997 males [54.7%]; *P* < .001) had variant *KRAS*. Adenocarcinomas were the most frequently diagnosed histology type, representing approximately 90% of tumors across age and *KRAS* status group (YO: 2086 patients with wild-type [90.9%] and 1390 patients with variant [89.9%] *KRAS*; LO: 9471 patients with wild-type [90.1%] and 6588 patients with variant [90.1%] *KRAS*). Grade II tumors were the most frequent grade, accounting for more than 50% of tumors across age groups (YO: 1359 patients with wild-type [59.2%] and 936 patients with variant [60.5%] *KRAS*; LO: 6173 patients with wild-type [58.7%] and 4556 patients with variant [62.3%] *KRAS*). A higher proportion of variant than wild-type *KRAS* tumors were seen for stage IV for YO (1025 patients [66.3%] vs 1421 patients [61.9%]; *P* = .03) and LO (4358 patients [59.6%] vs 5416 patients [51.5%]; *P* < .001) cancers (Table).

**Table.** Patient Social Demographic and Tumor Characteristics

Characteristic	Patients, No. (%) (N = 21 661)					
	YO^a^			LO^a^		
	*KRAS* wild type (n = 2296)	*KRAS* variant (n = 1546)	*P* value^b^	*KRAS* wild type (n = 10 508)	*KRAS* variant (n = 7311)	*P* value^b^
Age at diagnosis, mean (SD), y	41.69 (6.42)	42.38 (5.96)	.001	66.99 (10.64)	66.85 (10.46)	.37
Sex						
Female	979 (42.6)	807 (52.2)	<.001	4684 (44.6)	3314 (45.3)	.33
Male	1317 (57.4)	739 (47.8)		5824 (55.4)	3997 (54.7)	
Race and ethnicity						
Hispanic	388 (16.9)	277 (17.9)	<.001	988 (9.4)	818 (11.2)	<.001
Non-Hispanic American Indian, Alaska Native, or unknown	34 (1.5)	24 (1.6)		115 (1.1)	79 (1.1)	
Non-Hispanic Asian or Pacific Islander	257 (11.2)	126 (8.2)		796 (7.6)	504 (6.9)	
Non-Hispanic Black	274 (11.9)	250 (16.2)		1155 (11.0)	1088 (14.9)	
Non-Hispanic White	1343 (58.5)	869 (56.2)		7454 (70.9)	4822 (66.0)	
Insurance status						
Insured	1468 (63.9)	986 (63.8)	.84	6803 (64.7)	4655 (63.7)	.45
Medicaid	421 (18.3)	287 (18.6)		1416 (13.5)	1037 (14.2)	
Uninsured	150 (6.6)	110 (7.1)		378 (3.6)	264 (3.6)	
Unknown	257 (11.2)	163 (10.5)		1911 (18.2)	1355 (18.5)	
Sidedness						
Right sided	537 (23.4)	552 (35.7)	<.001	4271 (40.6)	3519 (48.1)	<.001
Left sided	1161 (50.6)	591 (38.2)		4049 (38.5)	2299 (31.4)	
Rectum	534 (23.3)	361 (23.4)		1821 (17.3)	1239 (16.9)	
Colon or NOS	64 (2.8)	42 (2.7)		367 (3.5)	254 (3.5)	
Histology						
Adenocarcinoma	2086 (90.9)	1390 (89.9)	.36	9471 (90.1)	6588 (90.1)	.98
Other	210 (9.1)	156 (10.1)		1037 (9.9)	723 (9.9)	
Grade						
Well differentiated, grade I	87 (3.8)	72 (4.7)	.01	487 (4.6)	410 (5.6)	<.001
Moderately differentiated, grade II	1359 (59.2)	936 (60.5)		6173 (58.7)	4556 (62.3)	
Poorly differentiated, grade III	481 (20.9)	266 (17.2)		2121 (20.2)	1092 (14.9)	
Undifferentiated or anaplastic, grade IV	103 (4.5)	59 (3.8)		502 (4.8)	217 (3.0)	
Unknown	266 (11.6)	213 (13.8)		1225 (11.7)	1036 (14.2)	
T stage						
T3	1355 (59.0)	859 (55.5)	.054	6504 (61.9)	4244 (58.1)	<.001
T4	662 (28.8)	465 (30.1)		2637 (25.1)	1982 (27.1)	
TX	279 (12.2)	222 (14.4)		1367 (13.0)	1085 (14.8)	
N stage						
N0	607 (26.5)	435 (28.1)	.02	4036 (38.4)	2644 (36.2)	.009
N1	1576 (68.6)	1007 (65.2)		5917 (56.3)	4254 (58.2)	
NX	113 (4.9)	104 (6.7)		555 (5.3)	413 (5.6)	
M stage						
M0	875 (38.1)	521 (33.7)	.001	5092 (48.4)	2953 (40.4)	<.001
M1a	711 (31.0)	457 (29.6)		2664 (25.4)	2097 (28.6)	
M1b	669 (29.1)	524 (33.9)		2523 (24.0)	2088 (28.6)	
M1X	41 (1.8)	44 (2.8)		229 (2.2)	173 (2.4)	
Stage						
0	7 (0.3)	3 (0.2)	.03	22 (0.2)	25 (0.3)	<.001
I	70 (3.0)	43 (2.8)		842 (8.0)	433 (5.9)	
II	192 (8.4)	117 (7.6)		1557 (14.8)	861 (11.8)	
III	595 (25.9)	343 (22.2)		2578 (24.5)	1564 (21.4)	
IV	1421 (61.9)	1025 (66.3)		5416 (51.5)	4358 (59.6)	
Unknown	11 (0.5)	15 (1.0)		93 (0.9)	70 (1.0)	
Year at diagnosis						
2010	323 (14.1)	206 (13.3)	.19	1385 (13.2)	921 (12.6)	.84
2011	381 (16.6)	258 (16.7)		1578 (15.0)	1089 (14.9)	
2012	378 (16.5)	249 (16.1)		1746 (16.6)	1223 (16.7)	
2013	382 (16.6)	260 (16.8)		1849 (17.6)	1288 (17.6)	
2014	459 (20.0)	276 (17.9)		2064 (19.6)	1434 (19.6)	
2015	373 (16.2)	297 (19.2)		1886 (17.9)	1356 (18.5)	
Vital status						
Colorectal cancer mortality	832 (36.2)	595 (38.5)	.18	3827 (36.4)	3059 (41.8)	<.001
Survival	1380 (60.1)	907 (58.7)		5827 (55.5)	3699 (50.6)	
Other or unknown mortality	84 (3.7)	44 (2.8)		854 (8.1)	553 (7.6)	

Abbreviations: LO, late onset; NOS, not otherwise specified; YO, young onset.

^a^ Patients were categorized as having YO cancer if diagnosed at ages 20 to 49 years and LO cancer if diagnosed at age 50 years or older.

^b^ *P* values compare *KRAS* wild type vs variant separately for YO and LO cancer.

### *KRAS* Distribution by Anatomical Site

**Figure 1.**
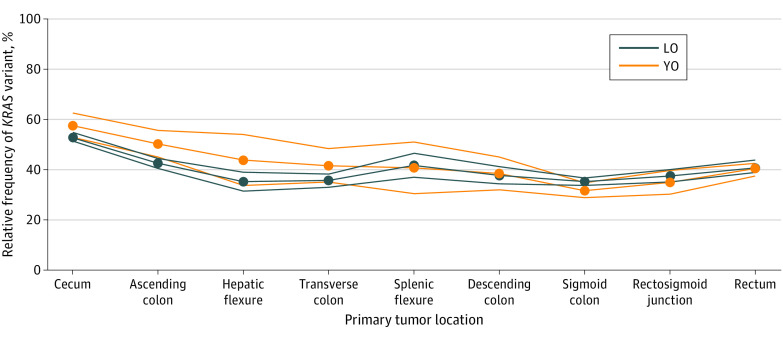
*KRAS* Variant Distribution by Anatomical Site LO indicates late onset; YO, young onset.

Regardless of age of onset, a tumor harboring a *KRAS* variant sequence was found to be more prevalent arising from the right compared with the left colon (Figure 1). As demonstrated in Figure 1, the relative frequency of a *KRAS* sequence variation appeared to diminish traversing from the proximal colon to the distal colon, with a greater increase in this frequency among YO tumors specifically.

### *KRAS* and CRC Mortality

**Figure 2.**
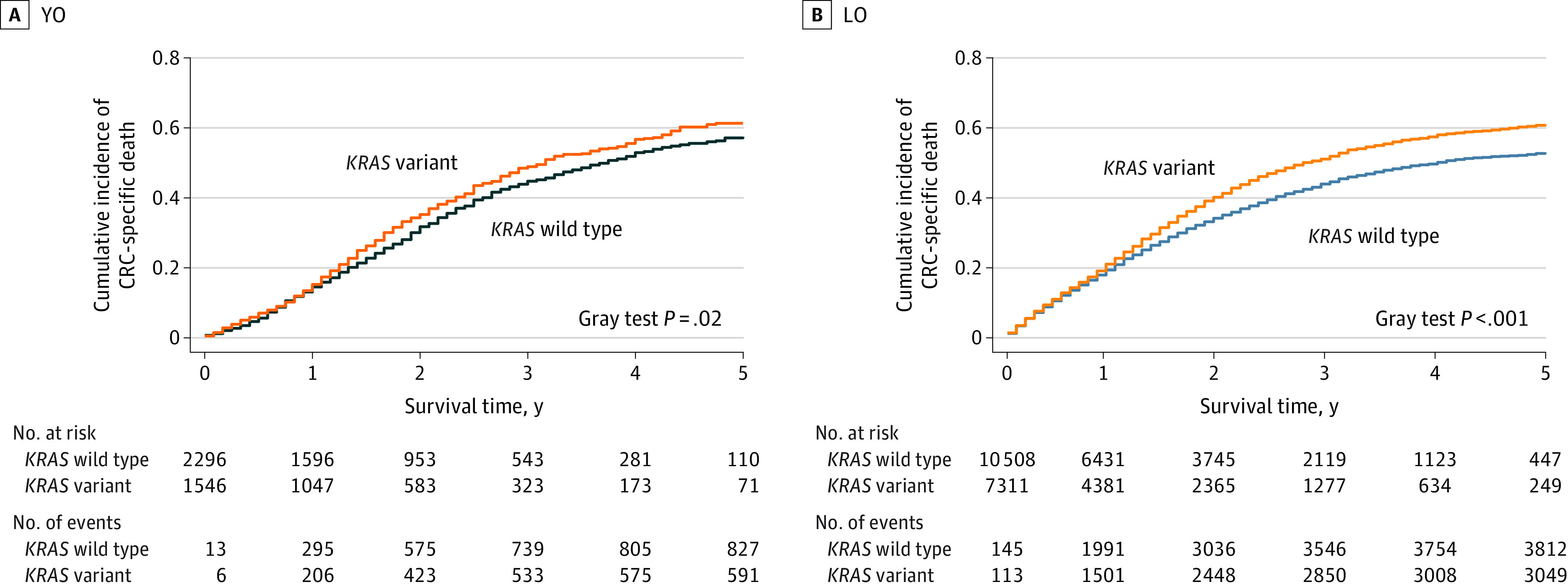
Cumulative Incidence of CRC (Colorectal Cancer) Death by *KRAS* Status Cumulative incidence of CRC death is presented in the presence of the competing risk of non-CRC death. LO indicates late onset; YO, young onset.

The cumulative incidence of CRC mortality was significantly higher for variant vs wild-type *KRAS* among patients with YO cancer (Gray test *P* = .02) (Figure 2A) and LO (Gray test *P* < .001) (Figure 2B). Consistent with these results, Kaplan-Meier curve and log-rank tests demonstrated lower a median CSS for patients with variant vs wild-type *KRAS* (YO: 3.0 years [95% CI, 2.8-3.3 years] vs 3.5 years [95% CI, 3.3-3.9 years]; *P* = .02; LO: 2.5 years [95% CI, 2.4-2.7 years] vs 3.4 years [95% CI, 3.3-3.6 years]; *P* < .001) (eFigure 1 in Supplement 1). The cumulative incidence of non CRC mortality in the presence of competing risk of CRC death among patients with YO and LO cancers is presented in eFigure 2 in Supplement 1.

**Figure 3.**
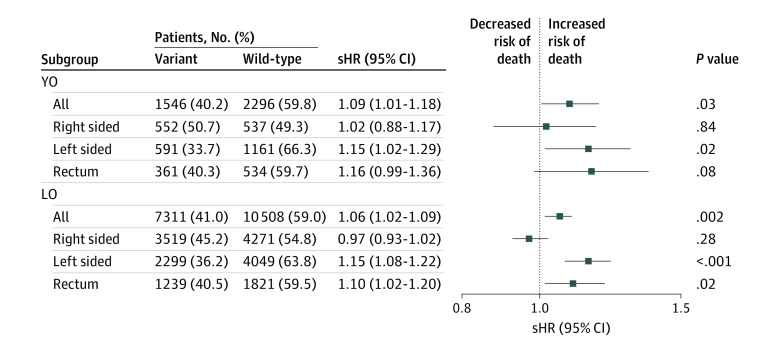
Multivariable Analyses for Colorectal Cancer–Specific Survival Performed Separately by Age of Onset Competing risk analyses were performed separately by age of onset and also under each age subgroub by tumor location to compare variant vs wild-type *KRAS* among patients with YO (young-onset; diagnosis at ages 20-49 years) and LO (late-onset; diagnosis at age ≥50 years) cancer. sHR indicates subdistribution hazard ratio.

Patients in the YO group with variant *KRAS* tumors showed significantly higher CSS subdistribution hazards for CRC-related death compared with patients with wild-type *KRAS* tumors (sHR, 1.09 [95%CI 1.01-1.18]; *P* = .03) (Figure 3). Mortality hazards increased by tumor location, from right (sHR, 1.02 [95% CI, 0.88-1.17]) to left (sHR, 1.15 [95% CI, 1.02-1.29]) and rectum (sHR, 1.16 [95% CI, 0.99-1.36]) (Figure 3). Similarly, patients in the LO group with variant *KRAS* showed significantly higher CSS for CRC-related death compared with patients with wild-type *KRAS* tumors (sHR, 1.06 [95% CI, 1.02-1.09]; *P* = .002) (Figure 3). However, comparisons of variant vs wild-type *KRAS* in the LO group showed no noticeable trend by tumor location from right (sHR, 0.97 [95% CI, 0.93-1.02]) to left (sHR, 1.15 [95% CI, 1.08-1.22]) and rectum (sHR, 1.10 [95% CI, 1.02-1.20]) (Figure 3). Comparable results were seen for analyses of CRC-related death within tumor stage and conducted separately for YO and LO groups (eFigures 3-6 in Supplement 1).

**Figure 4.**
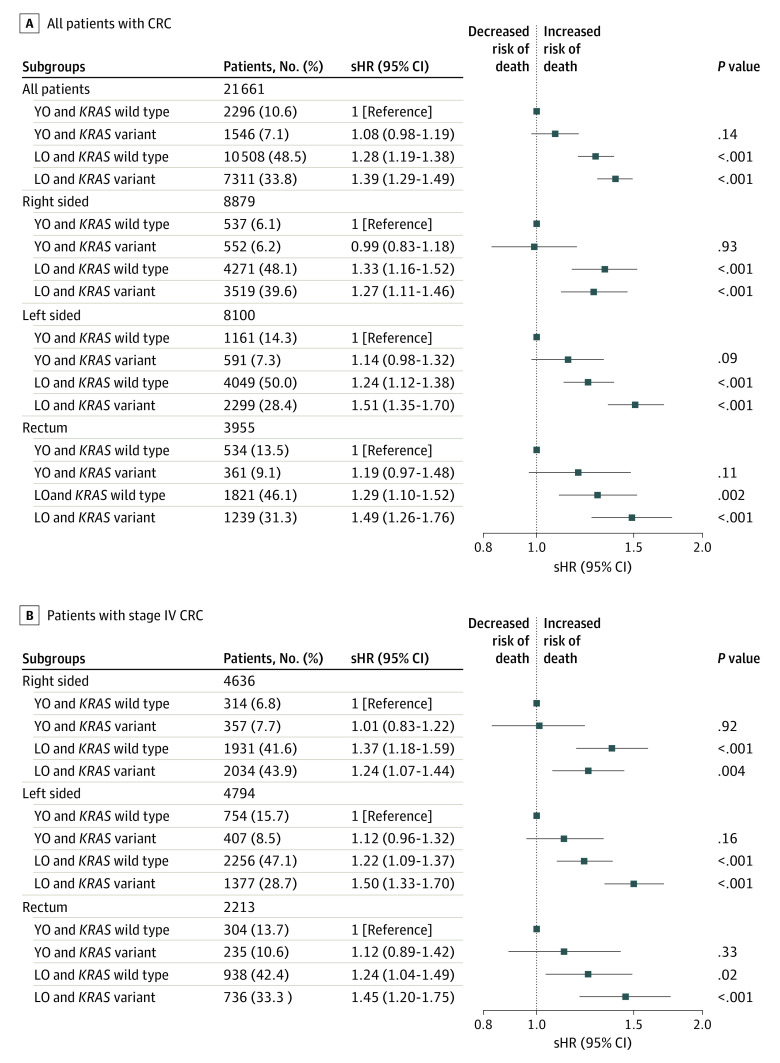
Multivariable Analyses for Colorectal Cancer (CRC)–Specific Survival by *KRAS* Status and Age at Onset Competing risk analyses were performed for CRC-specific survival to compare young-onset (YO) and *KRAS* variant, late-onset (LO) and *KRAS* wild-type, and LO and *KRAS* variant cancers with YO and *KRAS* wild-type cancer in each tumor location subgroup (all patients, right sided, left sided, and rectum) separately among all patients (A) and patients with stage IV CRC (B). sHR indicates subdistribution hazard ratio.

Finally, the assessment of the interaction between *KRAS* status and age using YO wild-type *KRAS* as the reference group did not reveal the presence of statistically significant additive interaction (Figure 4A). We found similar results when the analysis was limited to stage IV cancer (Figure 4B) and when analyses were stratified by tumor location (Figure 4A). These results were similar to those we found for CSS using Cox regression (eFigures 5 and 6 in Supplement 1).

## Discussion

Results from this cross-sectional study among 21 661 individuals with CRC indicated that *KRAS* sequence variation was associated with poorer survival among patients with YO and LO CRC. We also demonstrated that in YO, mortality hazards associated with *KRAS* sequence variation increased as tumor location became more distal. Our finding that variant *KRAS* was disproportionately located in right-sided tumors for LO was in concordance with 2 other recently published reports.^27,28^ However, for YO, *KRAS* sequence variation was more prevalent in the left, indicating a likely dominance of LO in published literature. Taken together, these data provide further detail into the association between *KRAS* sequence variation and clinical outcomes and may better characterize the association between *KRAS* status and age of CRC onset.

In this study, we present an opportunity to integrate some of the disparate information regarding *KRAS* status and prognosis. Our data suggest that the negative prognostic associations of a *KRAS* sequence variation were sustained among LO and YO CRC tumors, with a greater increase in hazards in YO CRC. This may in part provide a rationale for discrepancies in the existing literature regarding the degree to which *KRAS* sequence variation may be associated with clinical outcomes. Perhaps, the effect size of the association between a *KRAS* variant and clinical outcomes in earlier stage tumors was smaller given the high rates of cure. In contrast, the mortality rate among patients with metastatic cancer may be such that there was insufficient time to discriminate between clinical differences associated with the sequence variation.

Prognostic associations of variant *KRAS* in our study were limited to YO left-sided and rectal cancers. This outcome suggests that differences in outcomes observed compared with the previously cited studies (such as RASCAL^11^ vs RASCAL II^12^) may be attributed to differences in the distribution of anatomic sites in these studies. Given that our data were derived from a very large data set, drawing from a diverse population base included in the SEER database, we believe that results generated from our analysis may be broadly clinically applicable throughout the US. We also believe that our analysis comprising diagnoses from 2010 to 2015 was likely best representative of the state of the art of *KRAS* variant testing and incidence at this time. Previous analyses that failed to demonstrate associations of *KRAS* variants with clinical outcomes were uniformly from before 2009; since then, *KRAS* variant testing patterns have changed significantly, most likely driven by treatment implications. Furthermore, prior studies, including RASCAL^11^ and RASCAL II,^12^ did not take age at diagnosis (YO vs LO) or sidedness (right vs left) into consideration, which may account for the discrepancy between results of RASCAL and other previous studies and our results.^11,12^

### Limitations

There are several limitations to our study. First, the SEER database from which our analysis was derived lacks treatment information about specific types of medications. Because the presence of a *KRAS* variant had treatment implications and because clinical outcomes were necessarily dependent on the type of treatment received, this had the potential to impact the completeness of our data. However, given that the database was derived from years 2010 to 2015, a period of widespread use of *KRAS* status to guide treatment decisions, it was a reasonable assumption that most patients received appropriate therapy. Furthermore, given that *KRAS* status was available in only 21 661 of 202 237 total patients with CRC in our study, there may be a selection bias influencing the lack of testing, which may impact the generalizability of our results. In addition, *KRAS* is a new variable and was not routinely recorded, so it may have been incompletely captured by tumor registrars. Additionally, data that entered the SEER database may be impacted by systemic disparities in health care access, including availability of *KRAS* testing that may disproportionately affect minority racial and ethnic populations. Our data set also lacked specificity with regards to *KRAS* variant subtype, which has been demonstrated to carry implications for mortality and other outcomes, as described previously. This represents a potential missed opportunity to find greater changes in outcomes in associations described in this study, but it is unlikely to change overall conclusions we derived. Furthermore, data on the status of other molecular prognostic factors, such as microsatellite instability, *BRAF* V600E gene sequence variation, and human epidermal growth factor receptor 2 amplification, were not available from the SEER data set. These individual sequence variations are relatively uncommon, and we do not believe they would systemically alter results from this national, population-based registry.

## Conclusions

In this cross-sectional study, we aimed to further characterize the association of *KRAS* sequence variation with clinical outcomes by various demographic, clinical, and pathologic characteristics. We found that *KRAS* sequence variation was associated with negative prognostic outcomes in CRC, with greater increases in hazards for YO and distal tumor location. We believe that these findings may help to disentangle some existing discrepancies in the literature regarding *KRAS* status and clinical outcomes. Our analyses may bring some clarity to the matter via integration of our data with existing knowledge, as well as by adding nuance to the overall understanding of *KRAS* sequence variation and its clinical associations with age of onset and tumor sidedness.

## Notes

Supplement 1. **eFigure 1.** Kaplan-Meier Survival Curves for Cause-Specific Survival for Variant vs Wild-Type KRAS Among Patients With Young- and Late-Onset Colorectal Cancer **eFigure 2.** Cumulative Incidence of Non–Colorectal Cancer Death in the Presence of Competing Risk of Colorectal Cancer Death Among Patients With Young- and Late-Onset Cancer With KRAS Variant vs Wild Type **eFigure 3.** Forest Plot of Competing Risks Multivariable Analyses Performed Separately for Colorectal Cancer–Specific Survival to Compare Variant vs Wild-Type KRAS Among Patients With Young- and Late-Onset Cancer **eFigure 4.** Forest Plot of Competing Risks Multivariable Analyses Performed for Colorectal Cancer–Specific Survival to Compare KRAS Status and Age at Onset Among Patients With Colorectal Cancer **eFigure 5.** Forest Plot for Cause-Specific Mortality Hazards With 95% CIs for Variant vs Wild-Type KRAS Among Patients With Young- and Late-Onset Colorectal Cancer **eFigure 6.** Forest Plot for Cause-Specific Mortality Hazards With 95% CIs for KRAS Status and Age at Onset Among Patients With Colorectal Cancer

Supplement 2.
Data Sharing Statement

## References

[1] Siegel RL, Miller KD, Wagle NS, Jemal A. Cancer statistics, 2023. CA Cancer J Clin. 2023;73(1):17-48. doi:10.3322/caac.21763 36633525

[2] Wang W, Chen W, Lin J, Shen Q, Zhou X, Lin C. Incidence and characteristics of young-onset colorectal cancer in the United States: an analysis of SEER data collected from 1988 to 2013. Clin Res Hepatol Gastroenterol. 2019;43(2):208-215. doi:10.1016/j.clinre.2018.09.003 30686691

[3] Sinicrope FA. Increasing incidence of early-onset colorectal cancer. N Engl J Med. 2022;386(16):1547-1558. doi:10.1056/NEJMra2200869 35443109

[4] Willauer AN, Liu Y, Pereira AAL, . Clinical and molecular characterization of early-onset colorectal cancer. Cancer. 2019;125(12):2002-2010. doi:10.1002/cncr.31994 30854646 PMC6583775

[5] American Cancer Society. American Cancer Society guideline for colorectal cancer screening. Accessed April 15, 2021. https://www.cancer.org/cancer/types/colon-rectal-cancer/detection-diagnosis-staging/acs-recommendations.html

[6] Ahnen DJ, Wade SW, Jones WF, . The increasing incidence of young-onset colorectal cancer: a call to action. Mayo Clin Proc. 2014;89(2):216-224. doi:10.1016/j.mayocp.2013.09.006 24393412

[7] Lieu CH, Golemis EA, Serebriiskii IG, . Comprehensive genomic landscapes in early and later onset colorectal cancer. Clin Cancer Res. 2019;25(19):5852-5858. doi:10.1158/1078-0432.CCR-19-0899 31243121 PMC6774873

[8] Watson R, Liu TC, Ruzinova MB. High frequency of *KRAS* mutation in early onset colorectal adenocarcinoma: implications for pathogenesis. Hum Pathol. 2016;56:163-170. doi:10.1016/j.humpath.2016.06.010 27346571

[9] Sadough A, Afshari M, Rostami F, A systematic review and meta-analysis on the prevalence of *KRAS* gene mutation in samples of colorectal cancer. World Cancer Research Journal. 2020;7:e1522. doi:10.32113/wcrj_20203_1522

[10] Lièvre A, Bachet JB, Le Corre D, . *KRAS* mutation status is predictive of response to cetuximab therapy in colorectal cancer. Cancer Res. 2006;66(8):3992-3995. doi:10.1158/0008-5472.CAN-06-0191 16618717

[11] Andreyev HJ, Norman AR, Cunningham D, Oates JR, Clarke PA. Kirsten ras mutations in patients with colorectal cancer: the multicenter “RASCAL” study. J Natl Cancer Inst. 1998;90(9):675-684. doi:10.1093/jnci/90.9.675 9586664

[12] Andreyev HJN, Norman AR, Cunningham D, . Kirsten ras mutations in patients with colorectal cancer: the ‘RASCAL II’ study. Br J Cancer. 2001;85(5):692-696. doi:10.1054/bjoc.2001.1964 11531254 PMC2364126

[13] Ogino S, Meyerhardt JA, Irahara N, ; Cancer and Leukemia Group B; North Central Cancer Treatment Group; Canadian Cancer Society Research Institute; Southwest Oncology Group. *KRAS* mutation in stage III colon cancer and clinical outcome following intergroup trial CALGB 89803. Clin Cancer Res. 2009;15(23):7322-7329. doi:10.1158/1078-0432.CCR-09-1570 19934290 PMC2787689

[14] Roth AD, Tejpar S, Delorenzi M, . Prognostic role of *KRAS* and *BRAF* in stage II and III resected colon cancer: results of the translational study on the PETACC-3, EORTC 40993, SAKK 60-00 trial. J Clin Oncol. 2010;28(3):466-474. doi:10.1200/JCO.2009.23.3452 20008640

[15] Imamura Y, Morikawa T, Liao X, . Specific mutations in *KRAS* codons 12 and 13, and patient prognosis in 1075 *BRAF* wild-type colorectal cancers. Clin Cancer Res. 2012;18(17):4753-4763. doi:10.1158/1078-0432.CCR-11-3210 22753589 PMC3624899

[16] Blons H, Emile JF, Le Malicot K, . Prognostic value of *KRAS* mutations in stage III colon cancer: post hoc analysis of the PETACC8 phase III trial dataset. Ann Oncol. 2014;25(12):2378-2385. doi:10.1093/annonc/mdu464 25294886

[17] Yoon HH, Tougeron D, Shi Q, ; Alliance for Clinical Trials in Oncology. *KRAS* codon 12 and 13 mutations in relation to disease-free survival in *BRAF*-wild-type stage III colon cancers from an adjuvant chemotherapy trial (N0147 alliance). Clin Cancer Res. 2014;20(11):3033-3043. doi:10.1158/1078-0432.CCR-13-3140 24687927 PMC4040326

[18] Modest DP, Ricard I, Heinemann V, . Outcome according to *KRAS*-, *NRAS*- and *BRAF*-mutation as well as *KRAS* mutation variants: pooled analysis of five randomized trials in metastatic colorectal cancer by the AIO Colorectal Cancer Study Group. Ann Oncol. 2016;27(9):1746-1753. doi:10.1093/annonc/mdw261 27358379 PMC4999563

[19] Taieb J, Le Malicot K, Shi Q, . Prognostic value of *BRAF* and *KRAS* mutations in MSI and MSS stage III colon cancer. J Natl Cancer Inst. 2016;109(5):djw272. doi:10.1093/jnci/djw272 28040692 PMC6075212

[20] Gonsalves WI, Mahoney MR, Sargent DJ, ; Alliance for Clinical Trials in Oncology. Patient and tumor characteristics and *BRAF* and *KRAS* mutations in colon cancer, NCCTG/Alliance N0147. J Natl Cancer Inst. 2014;106(7):dju106. doi:10.1093/jnci/dju106 24925349 PMC4110470

[21] Charlton ME, Kahl AR, Greenbaum AA, . *KRAS* testing, tumor location, and survival in patients with stage IV colorectal cancer: SEER, 2010–2013. J Natl Compr Canc Netw. 2017;15(12):1484-1493. doi:10.6004/jnccn.2017.7011 29223986 PMC7458121

[22] Surveillance, Epidemiology, and End Results Program. SEER is an authoritative source for cancer statistics in the United States. National Cancer Institute. Accessed August 13, 2021. https://seer.cancer.gov/

[23] Fritz A, Percy C, Jack A, . International Classification of Diseases for Oncology. 3rd ed. World Health Organization; 2000.

[24] Surveillance, Epidemiology, and End Results Program. Race and Hispanic ethnicity changes. National Cancer Institute. Accessed October 1, 2023. https://seer.cancer.gov/seerstat/variables/seer/race_ethnicity/index.html

[25] Edge SB, Compton CC. The American Joint Committee on Cancer: the 7th edition of the AJCC Cancer Staging Manual and the future of TNM. Ann Surg Oncol. 2010;17(6):1471-1474. doi:10.1245/s10434-010-0985-4 20180029

[26] Fine JP, Gray RJ. A proportional hazards model for the subdistribution of a competing risk. J Am Stat Assoc. 1999;94(446):496-509. doi:10.1080/01621459.1999.10474144

[27] Loree JM, Pereira AAL, Lam M, . Classifying Colorectal cancer by tumor location rather than sidedness highlights a continuum in mutation profiles and consensus molecular subtypes. Clin Cancer Res. 2018;24(5):1062-1072. doi:10.1158/1078-0432.CCR-17-2484 29180604 PMC5844818

[28] Stintzing S, Tejpar S, Gibbs P, Thiebach L, Lenz HJ. Understanding the role of primary tumour localisation in colorectal cancer treatment and outcomes. Eur J Cancer. 2017;84:69-80. doi:10.1016/j.ejca.2017.07.016 28787661 PMC7505124

