# Supplementary material for: KRAS Sequence Variation as Prognostic Marker in Patients With Young- vs Late-Onset Colorectal Cancer
Source: JAMA Netw Open. 2023 Nov 30;6(11):e2345801. doi: 10.1001/jamanetworkopen.2023.45801 (PMC10690478; doi:10.1001/jamanetworkopen.2023.45801)
Supplement: Supplement 1. — eFigure 1. Kaplan-Meier Survival Curves for Cause-Specific Survival for Variant vs Wild-Type KRAS Among Patients With Young- and Late-Onset Colorectal Cancer eFigure 2. Cumulative Incidence of Non–Colorectal Cancer Death in the Presence of Competing Risk of Colorectal Cancer Death Among Patients With Young- and Late-Onset Cancer With KRAS Variant vs Wild Type eFigure 3. Forest Plot of Competing Risks Multivariable Analyses Performed Separately for Colorectal Cancer–Specific Survival to Compare Variant vs Wild-Type KRAS Among Patients With Young- and Late-Onset Cancer eFigure 4. Forest Plot of Competing Risks Multivariable Analyses Performed for Colorectal Cancer–Specific Survival to Compare KRAS Status and Age at Onset Among Patients With Colorectal Cancer eFigure 5. Forest Plot for Cause-Specific Mortality Hazards With 95% CIs for Variant vs Wild-Type KRAS Among Patients With Young- and Late-Onset Colorectal Cancer eFigure 6. Forest Plot for Cause-Specific Mortality Hazards With 95% CIs for KRAS Status and Age at Onset Among Patients With Colorectal Cancer [file jamanetwopen-e2345801-s001.pdf]

Supplemental Online Content

Aljehani MA, Bien J, Lee JSH, Fisher GA, Lin AY. *KRAS* sequence variation as prognostic marker in patients with young- vs late-onset colorectal cancer. *JAMA Netw Open*. 2023;6(11):e2345801. doi:10.1001/jamanetworkopen.2023.45801

eFigure 1. Kaplan-Meier Survival Curves for Cause-Specific Survival for Variant vs Wild-Type KRAS Among Patients With Young- and Late-Onset Colorectal Cancer
eFigure 2. Cumulative Incidence of Non–Colorectal Cancer Death in the Presence of Competing Risk of Colorectal Cancer Death Among Patients With Young- and Late-Onset Cancer With KRAS Variant vs Wild Type
eFigure 3. Forest Plot of Competing Risks Multivariable Analyses Performed Separately for Colorectal Cancer–Specific Survival to Compare Variant vs Wild-Type KRAS Among Patients With Young- and Late-Onset Cancer
eFigure 4. Forest Plot of Competing Risks Multivariable Analyses Performed for Colorectal Cancer–Specific Survival to Compare KRAS Status and Age at Onset Among Patients With Colorectal Cancer
eFigure 5. Forest Plot for Cause-Specific Mortality Hazards With 95% CIs for Variant vs Wild-Type KRAS Among Patients With Young- and Late-Onset Colorectal Cancer
eFigure 6. Forest Plot for Cause-Specific Mortality Hazards With 95% CIs for KRAS Status and Age at Onset Among Patients With Colorectal Cancer

This supplemental material has been provided by the authors to give readers additional information about their work.

© 2023 Aljehani M et al. *JAMA Network Open.*

eFigure 1. Kaplan-Meier Survival Curevs for Cause-Specific Survival for Variant vs Wild-Type *KRAS* Among Patients With Young- and Late-Onset Colorectal Cancer
YO

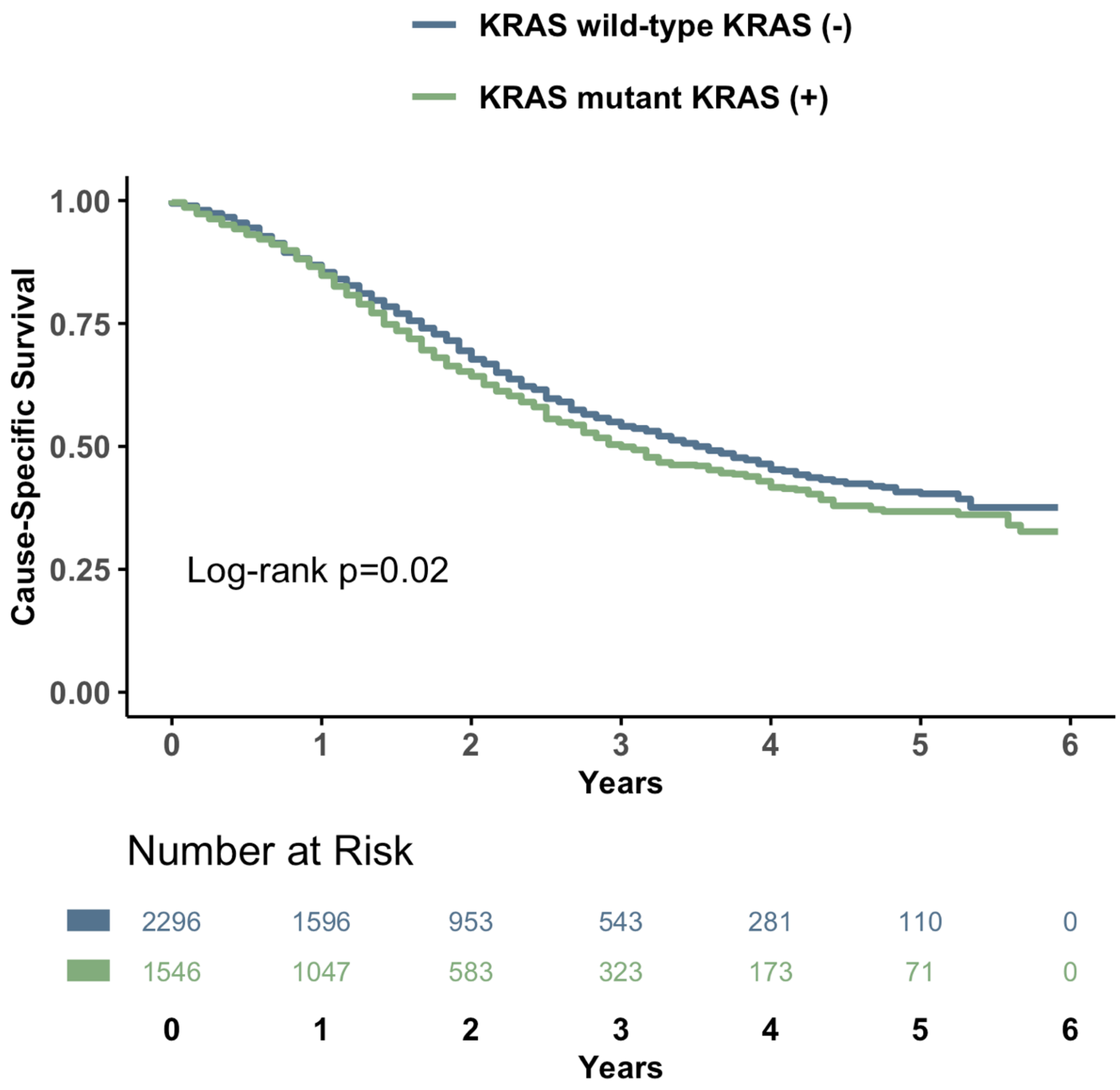

© 2023 Aljehani M et al. *JAMA Network Open.*

LO

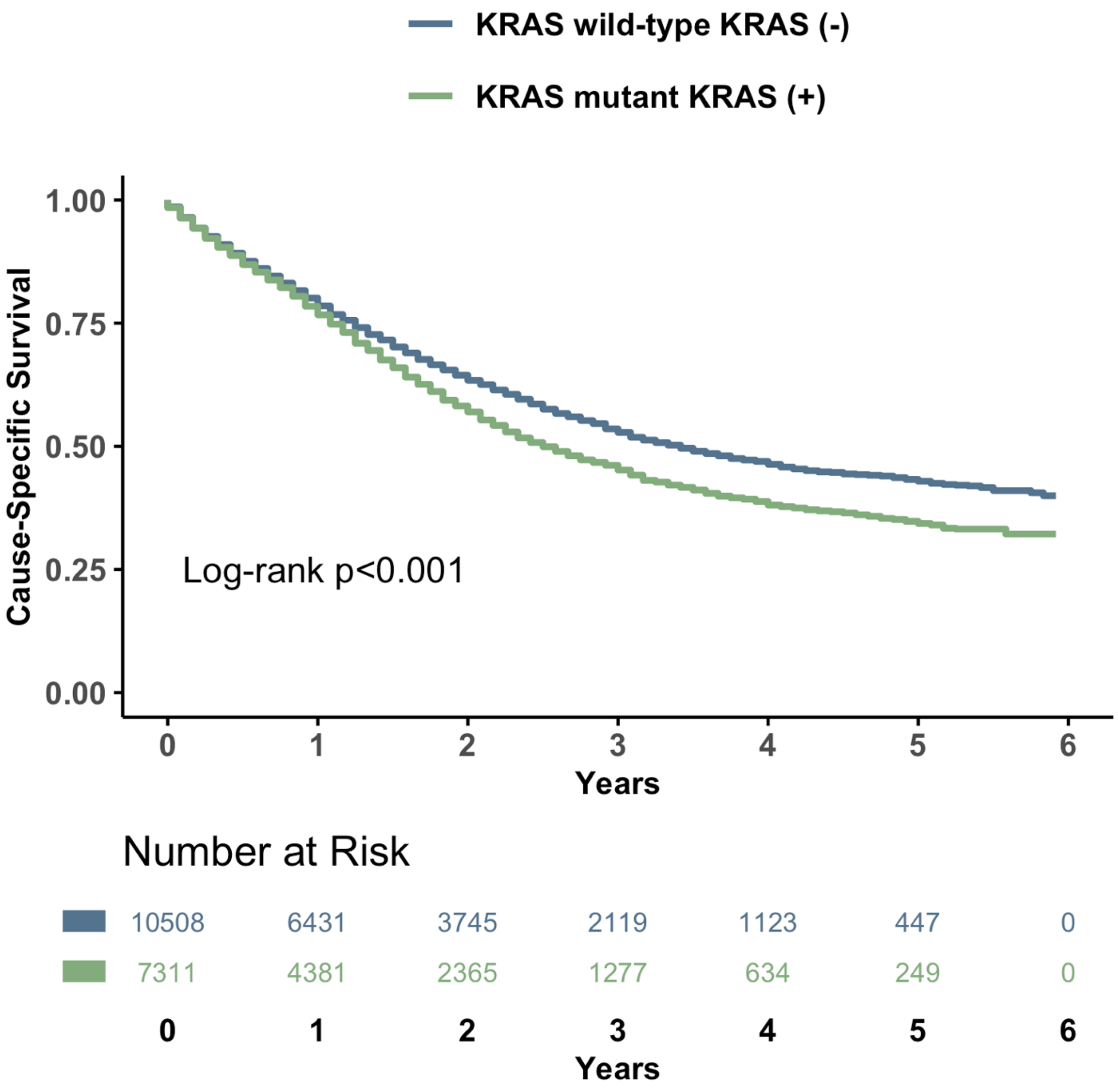

© 2023 Aljehani M et al. *JAMA Network Open.*

eFigure 2. Cumulative Incidence of Non–Colorectal Cancer Death in the Presence of Competing Risk of Colorectal Cancer Death Among Patients With Young- and Late-Onset Cancer With *KRAS* Variant vs Wild Type

YO

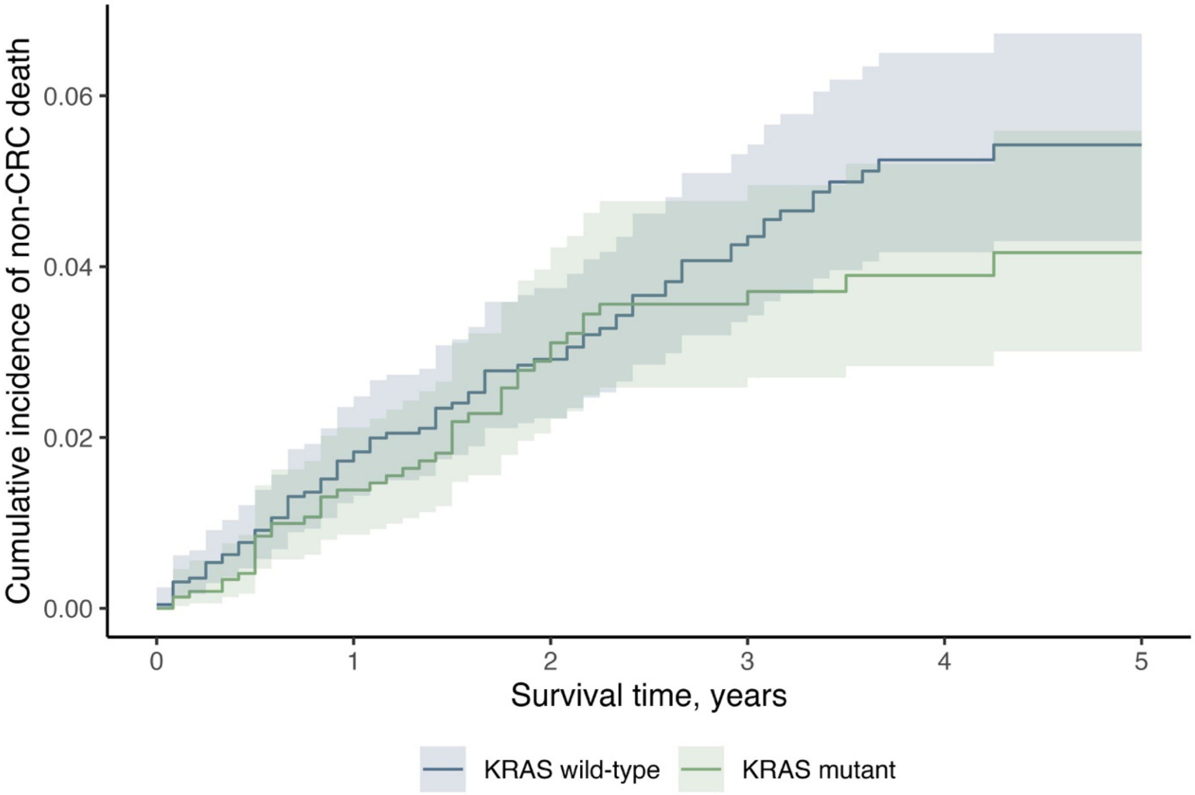

**At Risk**						
KRAS wild-type	2296	1596	953	543	281	110
KRAS mutant	1546	1047	583	323	173	71
**Events**						
KRAS wild-type	1	38	56	74	82	83
KRAS mutant	0	19	37	42	43	44

© 2023 Aljehani M et al. *JAMA Network Open.*

LO

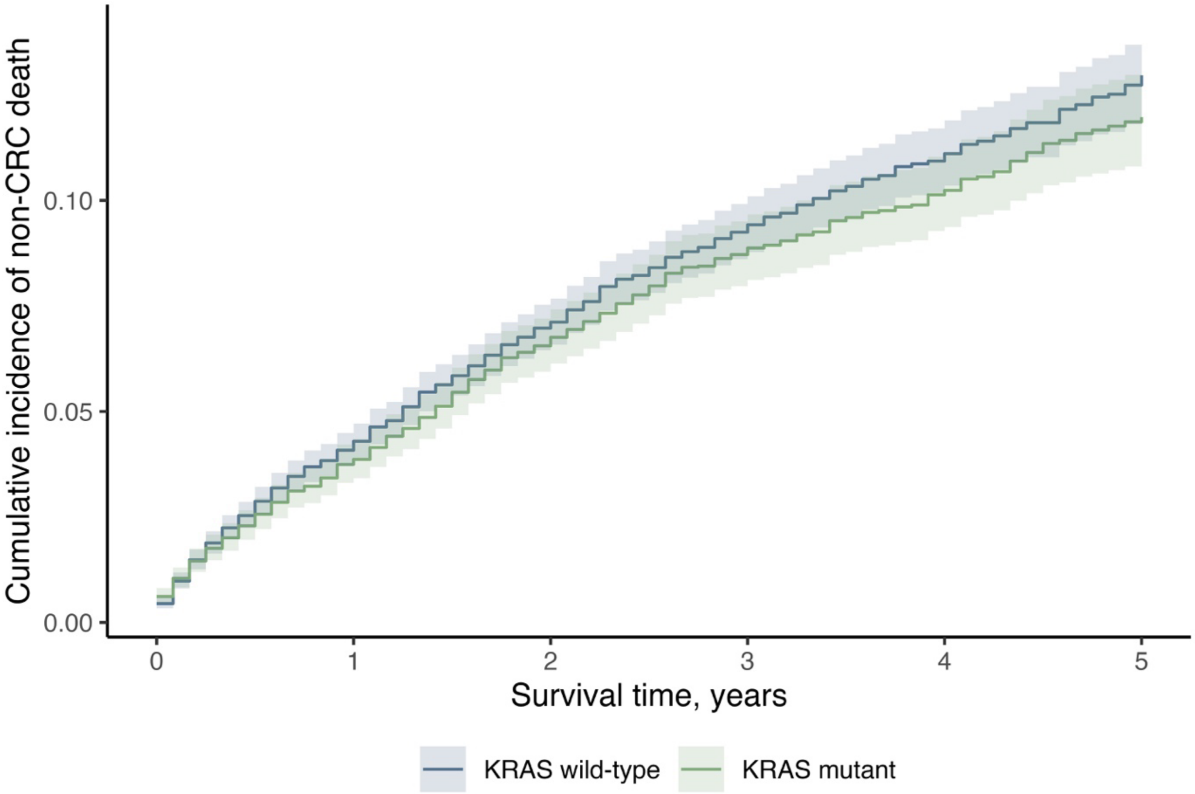

At Risk

	0	1	2	3	4	5
KRAS wild-type	10508	6431	3745	2119	1123	447
KRAS mutant	7311	4381	2365	1277	634	249

Events

	0	1	2	3	4	5
KRAS wild-type	47	414	623	749	811	847
KRAS mutant	45	260	408	488	522	547

© 2023 Aljehani M et al. *JAMA Network Open.*

eFigure 3. Forest Plot of Competing Risks Multivariable Analyses Performed Separately for Colorectal Cancer–Specific Survival to Compare Variant vs Wild-Type *KRAS* Among Patients With Young- and Late-Onset Cancer

Subgroups	KRAS Status N (%)		sHR (95%CI)	p-value
**Young-onset (20-49)**	**Mutant**	**Wild-type**		
Young-onset & Stage 0-II	163 (37.3)	269 (62.3)	1.27 (0.76-2.11)	0.36
Young-onset & Stage III	343 (36.6)	595 (63.4)	1.08 (0.87-1.35)	0.48
Young-onset & Stage IV	1,025 (41.9)	1,421 (58.1)	1.08 (1.0-1.18)	0.06
**Late-onset (50 +)**				
Late-onset & Stage 0-II	1,319 (35.3)	2,421 (64.7)	1.09 (0.95-1.25)	0.22
Late-onset & Stage III	1,564 (37.8)	2,578 (62.2)	1.04 (1.0-1.08)	0.05
Late-onset & Stage IV	4,358 (44.6)	5,416 (55.4)	1.04 (1.0-1.08)	0.05

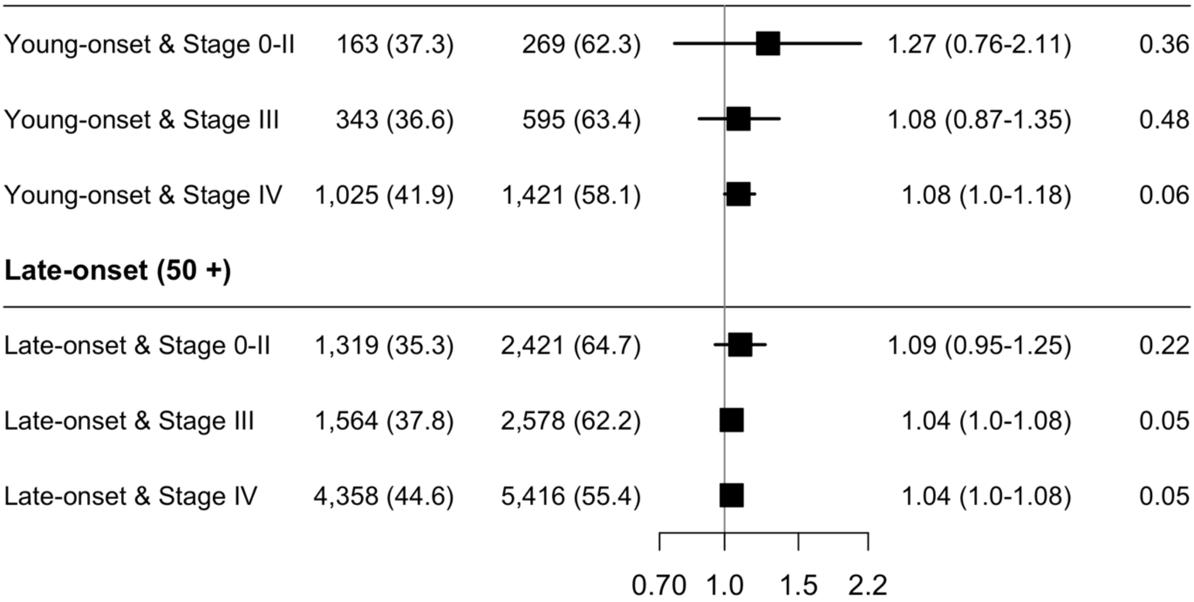

Subdistribution Hazards Ratio (sHR)

© 2023 Aljehani M et al. *JAMA Network Open.*

eFigure 4. Forest Plot of Competing Risks Multivariable Analyses Performed for Colorectal Cancer–Specific Survival to Compare *KRAS* Status and Age at Onset Among Patients With Colorectal Cancer

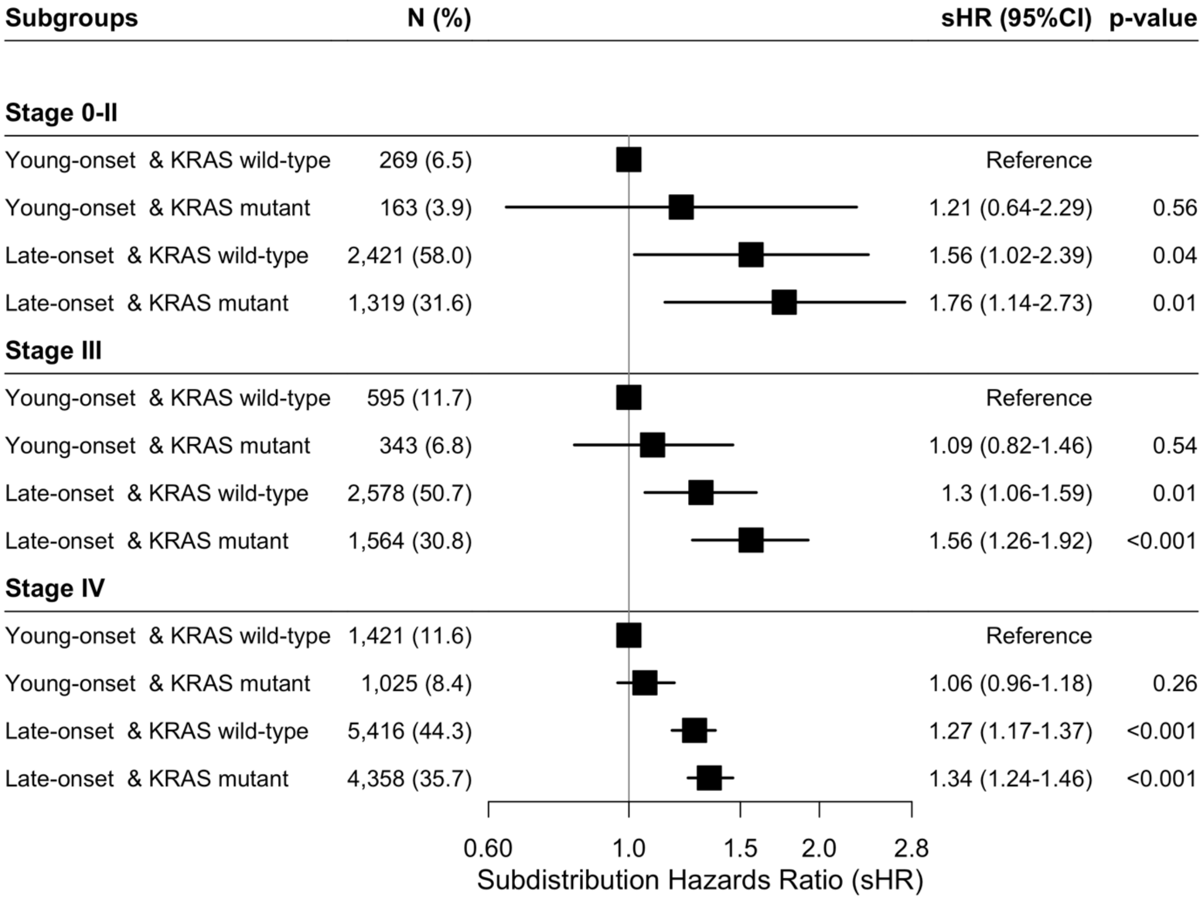

© 2023 Aljehani M et al. *JAMA Network Open.*

eFigure 5. Forest Plot for Cause-Specific Mortality Hazards With 95% CIs for Variant vs Wild-Type *KRAS* Among Patients With Young- and Late-Onset Colorectal Cancer

Groups	Deceased	Censored	HR (95%CI)	p-value
**KRAS mutant versus KRAS wild-type**				
**Young-onset (20-49)**	**1,427**	**2,415**		
All Young-onset Patients	1,427	2,415	1.07 (0.99-1.15)	0.09
Young-onset & Stage 0-II	38	394	1.18 (0.73-1.91)	0.488
Young-onset & Stage III	191	747	1.08 (0.87-1.33)	0.475
Young-onset & Stage IV	1,188	1,258	1.07 (0.98-1.16)	0.13
Young-onset & Right-sided	427	662	0.97 (0.85-1.11)	0.661
Young-onset & Left-sided	634	1,118	1.13 (1.00-1.27)	0.046
Young-onset & Rectum	313	582	1.13 (0.96-1.33)	0.141
**Late-onset (50 +)**	**6,886**	**10,933**		
All Late-onset Patients	6,886	10,933	1.06 (1.02-1.09)	0.017
Late-onset & Stage 0-II	436	3,304	1.09 (0.95-1.25)	0.227
Late-onset & Stage III	995	3,147	1.13 (1.03-1.24)	0.008
Late-onset & Stage IV	5,376	4,398	1.03 (0.99-1.07)	0.099
Late-onset & Right-sided	3,082	4,708	0.98 (0.93-1.03)	0.472
Late-onset & Left-sided	2,315	4,033	1.13 (1.06-1.19)	<0.001
Late-onset & Rectum	1,148	1,912	1.13 (1.04-1.23)	0.004

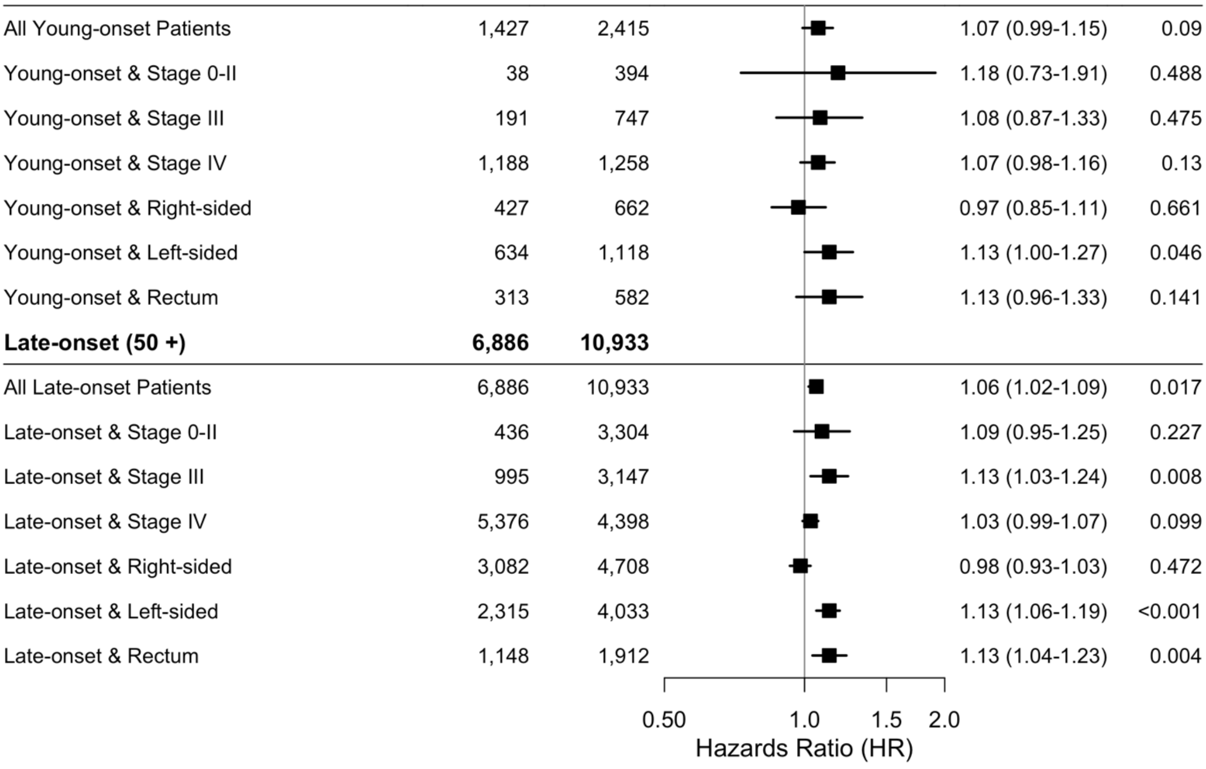

© 2023 Aljehani M et al. *JAMA Network Open.*

eFigure 6. Forest Plot for Cause-Specific Mortality Hazards With 95% CIs for *KRAS* Status and Age at Onset Among Patients With Colorectal Cancer

Groups	Deceased	Censored	HR (95%CI)	p-value
**Overall**	**8,313**	**13,348**		
Young-onset & KRAS wild-type	832	1,464	Reference	
Young-onset & KRAS mutant	595	951	1.06 (0.96-1.18)	0.244
Late-onset & KRAS wild-type	3,827	6,681	1.36 (1.26-1.47)	<0.001
Late-onset & KRAS mutant	3,059	4,252	1.46 (1.35-1.58)	<0.001
**Stage 0-II**	**474**	**3,698**		
Young-onset & KRAS wild-type	22	247	Reference	
Young-onset & KRAS mutant	16	147	1.22 (0.64-2.34)	0.541
Late-onset & KRAS wild-type	271	2,150	1.68 (1.08-2.6)	0.021
Late-onset & KRAS mutant	165	1,154	1.89 (1.2-2.96)	0.006
**Stage III**	**1,186**	**3,894**		
Young-onset & KRAS wild-type	113	482	Reference	
Young-onset & KRAS mutant	78	265	1.1 (0.82-1.46)	0.539
Late-onset & KRAS wild-type	595	1,983	1.35 (1.1-1.66)	0.004
Late-onset & KRAS mutant	400	1,164	1.62 (1.31-2)	<0.001
**Stage IV**	**6,564**	**5,656**		
Young-onset & KRAS wild-type	691	730	Reference	
Young-onset & KRAS mutant	497	528	1.06 (0.94-1.19)	0.357
Late-onset & KRAS wild-type	2,911	2,505	1.36 (1.25-1.48)	<0.001
Late-onset & KRAS mutant	2,465	1,893	1.43 (1.31-1.56)	<0.001
**Right-sided**	**3,509**	**5,370**		
Young-onset & KRAS wild-type	213	324	Reference	
Young-onset & KRAS mutant	214	338	0.96 (0.8-1.16)	0.694
Late-onset & KRAS wild-type	1,605	2,666	1.41 (1.22-1.63)	<0.001
Late-onset & KRAS mutant	1,477	2,042	1.33 (1.15-1.54)	<0.001
**Left-sided**	**2,949**	**5,151**		
Young-onset & KRAS wild-type	409	752	Reference	
Young-onset & KRAS mutant	225	366	1.13 (0.96-1.33)	0.134
Late-onset & KRAS wild-type	1,387	2,662	1.34 (1.2-1.49)	<0.001
Late-onset & KRAS mutant	928	1,371	1.64 (1.45-1.84)	<0.001
**Rectum**	**1,461**	**2,494**		
Young-onset & KRAS wild-type	173	361	Reference	
Young-onset & KRAS mutant	140	221	1.19 (0.95-1.49)	0.123
Late-onset & KRAS wild-type	637	1,184	1.33 (1.12-1.57)	<0.001
Late-onset & KRAS mutant	511	728	1.56 (1.31-1.85)	<0.001

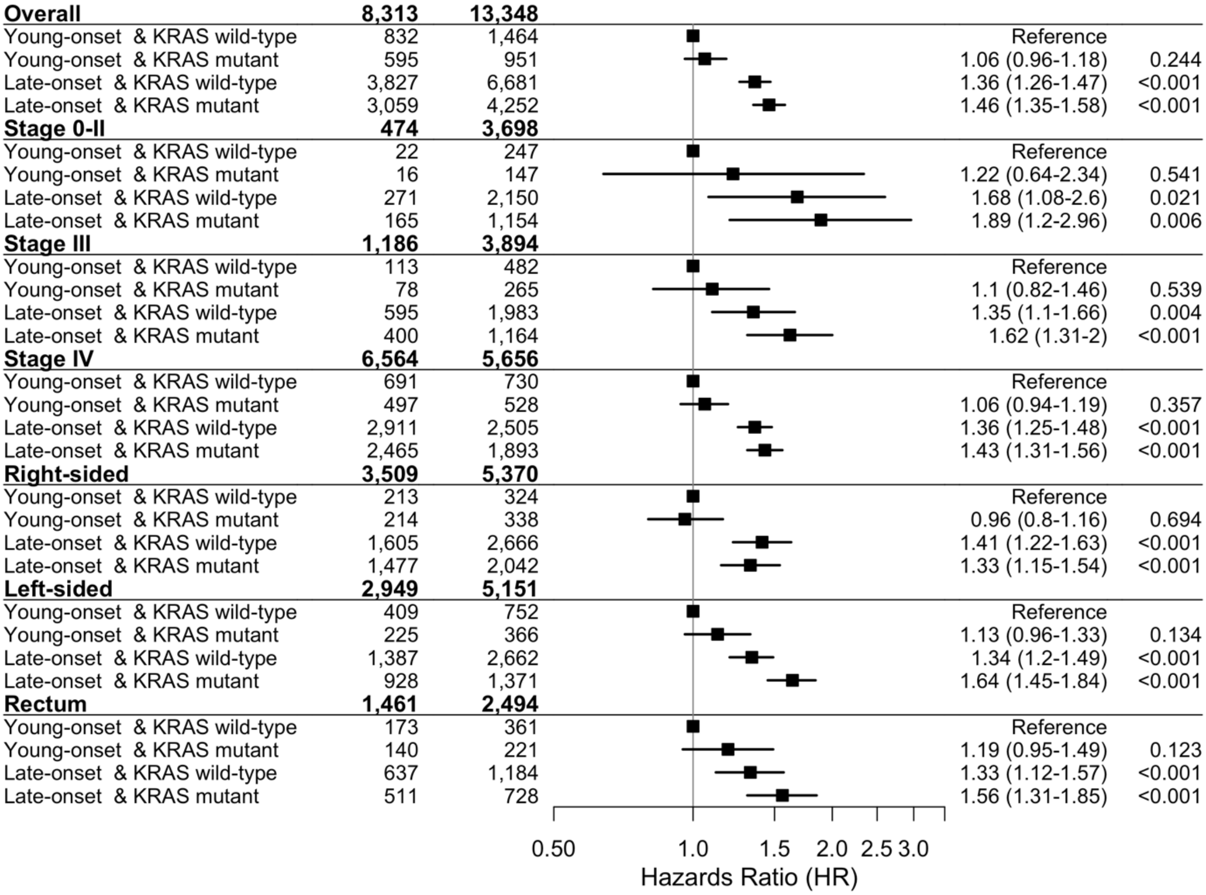

© 2023 Aljehani M et al. *JAMA Network Open.*
