# Supplementary material for: KRAS Sequence Variation as Prognostic Marker in Patients With Young- vs Late-Onset Colorectal Cancer
Source: JAMA Netw Open. 2023 Nov 30;6(11):e2345801. doi: 10.1001/jamanetworkopen.2023.45801 (PMC10690478; doi:10.1001/jamanetworkopen.2023.45801)
Supplement: Supplement 2. — Data Sharing Statement [file jamanetwopen-e2345801-s002.pdf]

Data Sharing Statement

Aljehani. KRAS Sequence Variation as Prognostic Marker in Patients With Young- vs Late-Onset Colorectal Cancer. *JAMA Netw Open*. Published online November 30, 2023. doi:10.1001/jamanetworkopen.2023.45801

Data
Data available: No
